# Brain response to prosodic boundary cues depends on boundary position

**DOI:** 10.3389/fpsyg.2013.00421

**Published:** 2013-07-18

**Authors:** Julia Holzgrefe, Caroline Wellmann, Caterina Petrone, Hubert Truckenbrodt, Barbara Höhle, Isabell Wartenburger

**Affiliations:** ^1^Department of Linguistics, Center of Excellence Cognitive Sciences, University of PotsdamPotsdam, Germany; ^2^Laboratoire Parole et Langage (LPL), UMR 7309, CNRS and Université Aix-MarseilleAix-en-Provence, France; ^3^Centre for General Linguistics (ZAS)Berlin, Germany

**Keywords:** prosodic boundaries, event-related potentials, closure positive shift, speech perception, prosody

## Abstract

Prosodic information is crucial for spoken language comprehension and especially for syntactic parsing, because prosodic cues guide the hearer's syntactic analysis. The time course and mechanisms of this interplay of prosody and syntax are not yet well-understood. In particular, there is an ongoing debate whether local prosodic cues are taken into account automatically or whether they are processed in relation to the global prosodic context in which they appear. The present study explores whether the perception of a prosodic boundary is affected by its position within an utterance. In an event-related potential (ERP) study we tested if the brain response evoked by the prosodic boundary differs when the boundary occurs early in a list of three names connected by conjunctions (i.e., after the first name) as compared to later in the utterance (i.e., after the second name). A *closure positive shift* (CPS)—marking the processing of a prosodic phrase boundary—was elicited for stimuli with a late boundary, but not for stimuli with an early boundary. This result is further evidence for an immediate integration of prosodic information into the parsing of an utterance. In addition, it shows that the processing of prosodic boundary cues depends on the previously processed information from the preceding prosodic context.

## Introduction

Listeners' comprehension of spoken language is guided by prosodic information provided in the uttered speech stream. Prosodic characteristics such as the distribution of pauses or changes in the fundamental frequency have an important structuring function and thus help the listener to understand the speaker's intention. Utterances are chunked into prosodic phrases of different strengths (e.g., Selkirk, 1980, 2011; Nespor and Vogel, 1986; Truckenbrodt, 2007), which helps to convey the correct meaning of a string of words. The boundaries of major prosodic phrases, so-called intonation phrases (Beckman and Pierrehumbert, 1986), are mainly signaled by three prosodic cues: a pitch change (i.e., a pitch rise or pitch fall indicates the presence of a boundary tone; this is usually followed by a pitch reset in the following phrase), final lengthening (i.e., an increase in the duration of the segments immediately preceding the boundary) and a pause (i.e., an interval of silence) between two phrases (see Peters et al., 2005 for German). Intonation phrase boundaries (IPBs) typically fall together with syntactic boundaries (Downing, 1970; Selkirk, 2005; for German: Truckenbrodt, 2005). For this reason, the perception of an IPB can be an important guide to the syntactic structure of spoken language; it is thus of special interest in psycholinguistic research in the attempt to bring to light how prosodic information is processed and how it contributes to sentence comprehension.

Numerous behavioral studies (see, amongst others, Price et al., 1991; Schafer, 1997; Kjelgaard and Speer, 1999; Carlson et al., 2001) have demonstrated an influence of prosodic boundary processing on syntactic analysis. Prosodic information is not present only at a local boundary, but instead unfolds throughout an utterance. Thus, the question arises of whether it is local boundary cues or rather prosodic information distributed across larger domains that has the primary influence on structural decisions during sentence processing. Proponents of the former view (e.g., Marcus and Hindle, 1990) have suggested that the processing of a prosodic boundary as a clue to syntactic structure is guided by the prosodic cues that occur in the direct vicinity of the boundary, regardless of other (prosodic) information that may be available to the listener. Prosodic boundary cues are thus supposed to be processed locally and context-independently. Others (e.g., Clifton et al., 2002) have argued against a solely local interpretation of prosodic boundaries. Instead, they provide evidence that the prosodic context in which an IPB occurs plays a major role, as the listener determines on a contextual basis whether a prosodic boundary is relevant for syntactic parsing decisions or not. In their work on the effect of prosodic information on the resolution of syntactic ambiguities, Clifton et al. (2002) identified two aspects that are relevant in this regard: the occurrence and the strength of neighboring prosodic boundaries, and the length of the prosodic phrase (e.g., the number of words or syllables) that precedes or follows a prosodic boundary.

Clifton et al. (2002) have found that listeners interpret a prosodic boundary relative to preceding boundaries or potential boundaries within the same utterance. For example, in sentences with attachment ambiguities like *Old men and women with very large houses* a preference for a high attachment of the modifier *with very large houses* was found more often when the boundary before the modifier had not been preceded by a boundary after *men*. Moreover, not the strength of the boundary *per se* had an effect on the attachment decisions but the strength of the boundary relative to the preceding one. Hence, the occurrence as well as the strength of a preceding boundary affected the perception of a subsequent boundary, as revealed by the listeners' parsing preferences. More recent evidence for this impact of relative prosodic boundary strength on the perception of prosodic boundaries comes from Snedeker and Casserly (2010), as well as Wagner and Crivellaro (2010).

Phrase length, that is the amount of material processed within one constituent, has been shown to affect prosodic phrasing in speech production and in the processing of implicit prosody in silent reading (Gee and Grosjean, 1983; Fodor, 1998; Watson and Gibson, 2004; Hwang and Schafer, 2009). Regarding the perception of prosodic boundaries, Clifton et al. (2006) demonstrate that phrase length affects the comprehension of syntactically ambiguous sentence structures. They presented participants in two auditory questionnaire experiments with sentences as in (1) and (2) (examples taken from Clifton et al. (2006); bracketing indicates the two different structures that were conveyed by prosodic phrasing):

1. (a) (Pat) or (Jay and Lee) convinced the bank president to extend the mortgage.
  (b) (Pat or Jay) (and Lee) convinced the bank president to extend the mortgage.
2. (a) (Patricia Jones) or (Jacqueline Frazier and Letitia Connolly) convinced the bank president to extend the mortgage.
  (b) (Patricia Jones or Jacqueline Frazier) and (Letitia Connolly) convinced the bank president to extend the mortgage.

The authors found a clear effect of the prosodic phrasing on sentence interpretation: participants were more likely to interpret stimuli in an “(X) or (Y and Z)” fashion when the prosodic phrasing suggests this analysis (examples 1a and 2a), while the “(X or Y) and (Z)” reading was favored for the stimuli with the correspondent prosodic phrasing (examples 1b and 2b.; see also Lehiste, 1973 for the effect of prosodic phrasing on the interpretation of this kind of stimuli). Crucially, the effect of prosody was significantly larger for stimuli with short constituents (1a and 1b) as compared to stimuli with long constituents (2a and 2b). Clifton and colleagues interpret this result by assuming that listeners treat the boundaries flanking short constituents as more informative for the syntactic analysis, because long constituents could also be flanked by a prosodic break to assure speech fluency.

These findings strongly suggest that globally distributed prosodic information is integrated into the processing of prosodic boundaries as markers of syntactic structure. However, based on the data so far it cannot be decided whether the global prosodic structure has a direct impact on the perception and processing of prosodic boundaries or whether the effects observed in the data by Clifton and colleagues occur later during the process of sentence interpretation. This shortcoming is due to the limitations that apply to off-line methods such as judgment and reaction time data. Here, on-line methods with a high temporal resolution, like event-related potentials (ERPs), are a useful tool to unravel the time course of a potential influence of the global prosodic structure on the perception of boundaries. Therefore, the present study uses ERPs to investigate the perception of prosodic boundary cues at different utterance positions, varying the phrase length and thereby the amount of contextual prosodic information given before an IPB occurs. To illustrate that ERPs are useful in addressing questions on the integration of prosodic information and, in particular, on the time course of prosodic phrase boundary processing, the following section briefly outlines previous ERP research on prosodic boundary processing.

Studies on sentence processing using ERPs demonstrate an early influence of prosodic information on the syntactic parsing process (see, e.g., Eckstein and Friederici, 2006). Considerable evidence for this stems from studies on the perception of prosodic boundaries. Steinhauer et al. (1999) conducted an ERP study in which they compared German sentences that contained either one or two IPBs. Brain responses to prosodic violations (here, a prosodic boundary inserted at a non-boundary position) showed that syntactic processing was misled by prosodic information at an early processing stage. Crucially, as a response to each IPB, the authors found a broadly distributed, large positive waveform. Because the ERP component coincides with the closure of major prosodic phrases, it has been termed closure positive shift (CPS). The CPS has been found to indicate the processing of prosodic boundaries in various languages: in German (e.g., Pannekamp et al., 2005; Männel and Friederici, 2009), English (e.g., Itzhak et al., 2010; Steinhauer et al., 2010; Pauker et al., 2011), Dutch (e.g., Kerkhofs et al., 2007; Bögels et al., 2010), Japanese (Wolff et al., 2008), Chinese (Li and Yang, 2009) and Korean (Hwang and Steinhauer, 2011, implicit prosody).

Pannekamp et al. (2005) presented participants with sentences comparable to the material used by Steinhauer et al. (1999). However, in addition to the natural condition, their stimulus material was systematically varied: experiments were carried out using jabberwocky sentences (stimuli without semantic content, but with appropriate use of functional morphemes), pseudo-word sentences (containing neither semantic nor syntactic information), and hummed speech (without segmental information). Although the scalp distribution varied for the conditions that provided less linguistic information, the CPS was elicited in all four conditions. This shows that the CPS component occurs independently of semantic, syntactic, or segmental information. Moreover, Steinhauer et al. (1999) as well as Männel and Friederici (2009) demonstrate that a pause between two intonation phrases is not necessary to elicit a CPS. Hence, the CPS is not a variant of early auditory evoked potentials that signals the detection of new auditory input (e.g., after a break) and does thus not reflect lower level acoustic processing.

Concerning the impact of contextual prosodic information on prosodic phrasing, Hwang and Steinhauer (2011) used the CPS to demonstrate an influence of phrase length on (implicit) prosodic phrasing (which had previously been shown in behavioral production studies, see above). In a silent reading study on Korean they found that only longer sentence-initial constituents elicited a CPS, while no effect was found for short subject noun phrases. Here, the CPS was considered to reflect the subvocal generation of an additional prosodic boundary, which was only triggered by long constituents.

To summarize, ERP studies firstly support the notion of an early integration of prosodic information in general. Secondly, they provide converging evidence that the CPS component reflects prosodic boundary processing, as the occurrence of the CPS depends on prosodic information in the speech input, that is, the prosodic cues that mark the closure of an IPB, but not on other linguistic information or on mere acoustic changes in the input. Furthermore, the CPS has been shown to be sensitive to contextual effects on prosodic phrasing in silent reading.

Based on these findings the present study makes use of the CPS as an indicator of IPB processing in differing prosodic contexts. In contrast to the complex sentences used in previous studies, our study employed coordinated lists with different syntactic and semantic subgroupings of the elements. The production of prosodic boundary cues in such subgroupings is shown in Ladd (1988) and Féry and Truckenbrodt (2005)—where the lists are lists of sentences—and in Wagner (2005) and Kentner and Féry (2013), where the lists are lists of names as in our experiment. We varied the position of the utterance-internal IPB in our stimulus material to determine whether prosodic context affects the processing of boundary cues as reflected by the occurrence of the CPS. We compared stimuli that contained an early IPB, that is, after a short intonation phrase, with stimuli that contained a late IPB, that is, an IPB preceded by a larger amount of prosodically structured material. Crucially, the local acoustic markers of the prosodic boundaries did not differ across the two positions. The reasoning goes as follows: If an IPB is processed solely based on the local occurrence of specific acoustic cues in the signal, we would expect the positive deflection to occur in both conditions with the latency of the component varying as a function of the boundary position (early vs. late). If, in contrast, IPB processing is affected by the boundary position, we should see differences in the occurrence of the CPS between the two conditions that go well-beyond the presupposed latency difference.

## Materials and methods

### Participants

Eighteen students of the University of Potsdam (12 women, age range: 20–28 years, mean age: 24.0 years) participated after giving informed consent. They were native speakers of German with no reported hearing or neurological disorders. All participants were right-handed, as assessed by a German version of the Edinburgh Handedness Inventory (Oldfield, 1971), and received course credits or reimbursement for their participation.

### Material

Each experimental item consisted of a list of three disyllabic, trochaic names that were connected by *oder* (or) and *und* (and). There were two experimental conditions that differed with respect to the prosodic grouping of the three names: in (a), the EARLY condition, an IPB—signaled by a pitch change, final lengthening and a pause—occurred after the first name, while in (b), the LATE condition, the IPB occurred after the second name of the list (the position of the IPB is indicated by a hash mark in the examples):

3. (a) EARLY condition:[Mona]_IP_ # [oder Lena und Lola]_IP_.
  (b) LATE condition: [Mona oder Lena]_IP_ # [und Lola]_IP_.

Six German names (*Lola, Lena, Lilli, Manu, Mona, Nina*) were used to construct six different lists of three names. Hence, not all possible combinations of names were used, but it was ensured that each name occurred once in the first, the second, and the utterance-final position. All names were composed of four sonorants to allow a thorough acoustic analysis of the experimental material (see below). The six different lists of names were recorded in both prosodic conditions. During the experiment, each of these items was presented ten times, yielding a total of 60 experimental items per condition.

The stimuli were recorded in an anechoic booth by a naïve female native speaker of German. To ensure that the speaker produced the name sequences with the intended prosodic structure (early vs. late IPB), she was provided with a written list of the stimuli in which the intended prosodic grouping was indicated by bracketing, that is *(Mona) (oder Lena und Lola)* for the EARLY condition and *(Mona oder Lena) (und Lola)* for the LATE condition. Each stimulus was preceded by the same context question (*Wer kommt?* “Who is coming?”) read by the experimenter. The speaker was instructed to read the name triples in such a way that the experimenter (who could not see the speaker's text) was able to mark the indicated grouping by adding the brackets in her written version of the stimulus list.

**Figure 1:**
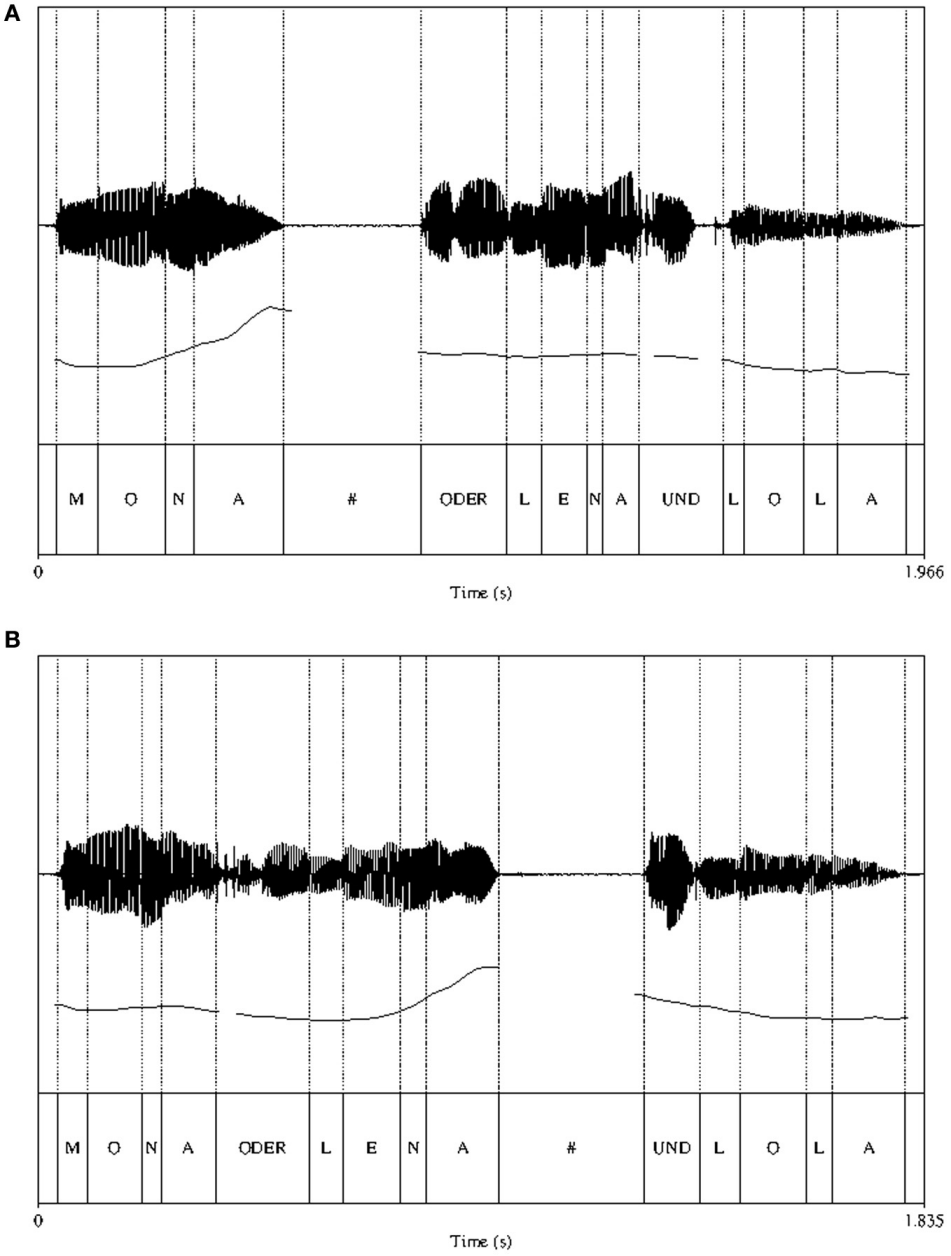
**Spectrogram, pitch track and segmental labeling for the EARLY (panel A) and LATE (panel B) IPB conditions**. Dotted lines mark the segmental boundaries. The silent pause after the IP boundary is indicated by a hash mark.

An example of a typical minimal pair is displayed in Figure 1. The figure shows that in both EARLY and LATE conditions the IPB is signaled by the presence of a silent pause (marked by a hash mark in the segmental labeling tier), a pitch change (instantiated as a pitch rise and a reset after the pause) and a lengthening of the preboundary segment.

Acoustic analyses were carried out with Praat (Boersma and Weenink, 2010) to confirm that the relevant boundary cues—pitch change, final lengthening and pause—were present and that the items from both conditions only differed in the critical respect, that is, the position of the prosodic boundary. An overview of the results is given in Table 1.

**Table 1:** **Mean acoustic correlates of prosodic cues in the experimental stimuli**.

**Acoustic correlate**	**EARLY condition**		**LATE condition**	
	**First name**	**Second name**	**First name**	**Second name**
Pitch rise in Hz (*SD)*	**144 (21)**	31 (23)	26 (16)	**151 (21)**
Maximum pitch in Hz (*SD*)	**350 (21)**	263 (23)	241 (20)	**340 (19)**
Final vowel duration in ms (*SD*)	**172 (21)**	84 (14)	113 (13)	**152 (17)**
Pause duration in ms (*SD*)	**297 (29)**	–	–	**268 (20)**

Numbers in bold represent measures from the IPB present in the respective condition.

The durational properties—pause and final lengthening—were assessed by measuring the length of the final vowel of the first and the second name, as well as the length of a possible subsequent pause. In the EARLY condition, the final vowel duration of the first name was more than twice as long as on the second name and was followed by an extended pause, whereas no pause occurred after the second name. In the LATE condition we observed the reversed pattern: the mean final vowel duration of the first name was shorter than the final vowel duration of the second name, which was again followed by a pause.

To assess the pitch change we measured the preboundary pitch rise which occurred on the names that were potentially followed by an IPB. Therefore, the minimum of the fundamental frequency on the first sonorant of each first and second name was measured as well as the maximum of the fundamental frequency on the final vowel (i.e., a high boundary tone). The difference of these values was used to calculate the pitch rise preceding the potential boundary position. In the EARLY condition, a major pitch rise occurred at the early boundary position: The pitch rise on the first name was almost five times as large as the slight rise measured on the second name (with no subsequent IPB). In the LATE condition, a comparably large pitch rise was observed on the second name, again in contrast to only a slight pitch rise on the first name.

The slight but perceivable pitch rise at the non-IPB positions (i.e., the second name in the EARLY condition and the first name in the LATE condition, see Table 1) hints at the presence of different tonal events at these positions. In particular, according to Truckenbrodt (2007), it is attributed to the presence of a pitch accent and an edge tone of the accent domain. Such domain is the Accentual Phrase (AP), which is a prosodic constituent lower than the IP. Hence, each prosodic word in our material constitutes an AP:

4. (a) EARLY condition:[(Mona)_AP_]_IP_ # [(oder Lena)_AP_ (und Lola)_AP_]_IP_.
  (b) LATE condition: [(Mona)_AP_ (oder Lena)_AP_]_IP_ # [(und Lola_AP_)]_IP_.

In sum, the acoustic analyses confirmed that the relevant IPB cues were present and did not differ in strength between conditions. There was only a positional difference: in the EARLY condition the crucial prosodic boundary cues—pitch change, final lengthening and pause—were present at the end of the first name, while in the LATE condition they occurred at the end of the second name in the sequence. Taking the offset of the name before the IPB as indication of the IPB position, the positional difference amounts to ~500 ms: in the EARLY condition, the first name ends on average 488 ms (*SD* = 57 ms) after stimulus onset, while the second name in the LATE condition ends on average 992 ms (*SD* = 49 ms) after stimulus onset. Since latency differences—even if intended in the experimental design of stimulus material—play an important role in the interpretation of grand average ERPs, duration measures for the critical utterance parts in the experimental material are presented in Table 2. It becomes obvious that the stimuli systematically differ in critical word durations (that is, due to final lengthening noun phrases are longer at IPB positions than at non-boundary positions) but not, for example, in total length. Moreover, latency differences occur between conditions, whereas duration measures within conditions are relatively homogenous.

**Table 2:** **Duration in ms for critical words, pauses and utterance parts (before/after the pause), rounded to the nearest whole number, for each token employed in the EARLY condition (EA1–EA6) and in the LATE condition (LA1–LA6), respectively**.

**Experimental stimulus**	**Duration for critical words in ms**				**Duration for utterance parts in ms**			
	**NP1**	**Conj1**	**NP2**	**Conj2 + NP3**	**First part**	**Pause**	**Second part**	**Total**
**EARLY CONDITION**								
EA1 [Mona] [oder Lena und Lola]	545	189	287	639	545	306	1115	1966
EA2 [Lena] [oder Lola und Mona]	496	172	315	618	496	311	1104	1911
EA3 [Lola] [oder Mona und Lena]	509	160	274	547	509	273	981	1763
EA4 [Nina] [oder Lilli und Manu]	472	157	261	631	472	322	1048	1842
EA5 [Lilli] [oder Manu und Nina]	383	179	320	609	383	318	1108	1808
EA6 [Manu] [oder Nina und Lilli]	522	167	292	461	522	250	921	1693
Mean	488	171	291	584	488	297	1046	1830
*SD*	57	12	23	68	57	29	80	99
**LATE CONDITION**								
LA1 [Mona oder Lena] [und Lola]	368	193	397	580	958	297	580	1835
LA2 [Lena oder Lola] [und Mona]	434	181	422	629	1037	259	629	1924
LA3 [Lola oder Mona] [und Lena]	382	228	402	568	1012	277	568	1857
LA4 [Nina oder Lilli] [und Manu]	362	196	352	611	910	269	611	1790
LA5 [Lilli oder Manu] [und Nina]	392	194	418	579	1004	267	579	1851
LA6 [Manu oder Nina] [und Lilli]	397	227	408	525	1031	237	525	1793
Mean	389	203	400	582	992	268	582	1842
*SD*	26	20	25	36	49	20	36	49

Note that for the EARLY condition duration of NP1 is identical to the first part of the utterance and for the LATE condition duration of Conj2 + NP3 is identical to the second utterance part.

### Procedure

The 120 experimental items were presented aurally (using E-A-RTONE 3A Insert Earphones, Aearo Technologies Auditory Systems, Indianapolis, USA) in a pseudo-randomized order with an inter-stimulus-interval of 4000 ms. The same sequence of names never occurred in consecutive trials and at most three consecutive trials belonged to the same condition. Participants were instructed to listen carefully and to avoid eye blinking and other body movements during stimulus presentation. To minimize eye movements, a fixation cross was displayed in the center of a monitor starting 1500 ms before stimulus onset until the end of the respective trial. The experiment lasted ~12 min.

The electroencephalogram (EEG) was continuously recorded from 30 cap-mounted active Ag/AgCl electrodes (Brain Products, Gilching, Germany) with a sampling rate of 1000 Hz. Electrodes were placed into the EEG cap at the following positions: Fp1/2, F7/8, F5/6, F3/4, Fz, FC3/4, FCz, T7/8, C3/4, C5/6, Cz, CP3/4, CPz, P7/8, P3/4, Pz, POz, O1/2. The electrooculogram (EOG) was recorded from electrodes placed above and below the right eye. Impedances were kept below 5 kΩ. The EEG recording was referenced on-line to the left mastoid and re-referenced off-line to linked mastoid electrodes.

### Data analysis

The EEG data were analyzed using Brain Vision Analyzer (version 2.01; Brain Products, Gilching, Germany). A digital band pass filter ranging from 0.2 to 70 Hz was applied to remove very slow drifts and muscle artifacts, and we also applied a 50 Hz notch filter. Epochs of 2200 ms, relative to stimulus onset, were extracted from the continuous EEG signal. Eye blinks and eye movements in the epochs were corrected by a computer algorithm (Gratton et al., 1983). All other artifacts were detected manually and contaminated segments were excluded from further analysis. The mean number of averaged trials per subject was 51.9 for the EARLY condition (*SD* = 5.4; 86.5%) and 52.1 for the LATE condition (*SD* = 5.3; 86.8%). The data of three additional participants were excluded from further analysis because the criterion of at least 40 artifact-free trials per condition (67%) was not met.

Two types of statistical analyses were performed that have been applied before to quantify CPS effects: First, we conducted analyses time-locked to the stimulus onset with a prestimulus baseline of 200 ms (see, e. g., ERP analyses in Steinhauer et al., 1999; Pannekamp et al., 2005; Männel and Friederici, 2009, 2011), as well as adjusted to a baseline from 200 to 400 ms after stimulus onset, covering the early onset components. Second, additional analyses relative to potential boundary positions within the stimuli were conducted (see Kerkhofs et al., 2007; Bögels et al., 2010; Pauker et al., 2011, for comparable analyses), that is, time-locked to the offset of the first and the second name. In both cases, separate analyses were applied to lateral and midline electrodes. The following electrodes were used in the statistical analysis of lateral sites and were—by crossing the factors Region (anterior vs. central vs. posterior) and Hemisphere (left vs. right)—subdivided into six regions of interest: left anterior (F3, F7), right anterior (F4, F8), left central (FC3, C3), right central (FC4, C4), left posterior (CP3, P3), and right posterior (CP4, P4). In contrast, the separate analysis of the midline electrodes contained four levels of the factor electrode (Fz vs. FCz vs. Cz vs. Pz).

## Results

### Analyses relative to stimulus onset

#### Descriptive results

**Figure 2:**
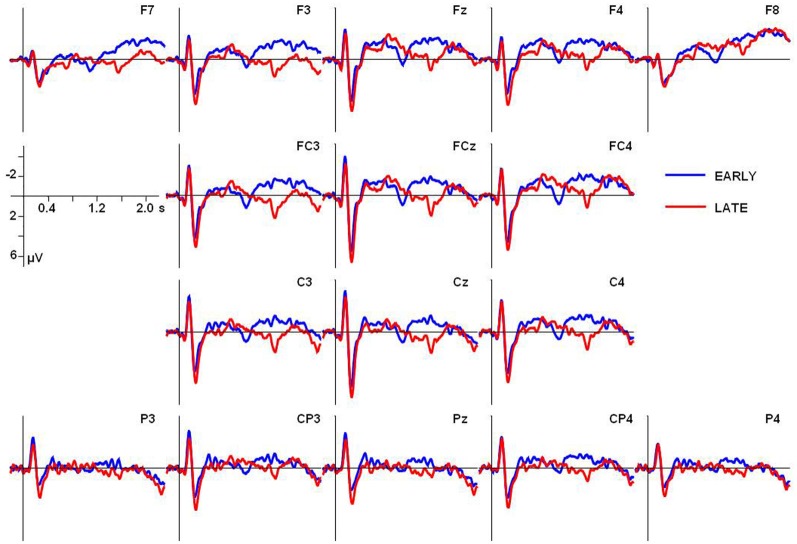
**Grand average ERPs adjusted to a prestimulus baseline of 200 ms for both conditions at the electrodes used in the statistical analyses**. In all ERP figures an 8-Hz low-pass Butterworth zero-phase filter was applied off-line only for presentation purposes; all statistical analyses were performed on unfiltered data.

**Figure 3:**
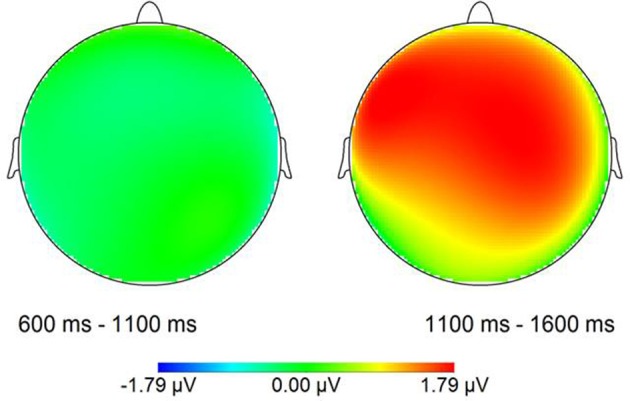
**Voltage maps of difference waves (adjusted to a prestimulus baseline of 200 ms) for the critical time windows used in the first statistical analysis relative to stimulus onset (see below for details)**.

The grand average ERP waves adjusted to a prestimulus baseline of 200 ms at the 16 electrodes used in the statistical analyses are illustrated in Figure 2. Additionally, voltage maps of differences waves based on all electrodes are shown in Figure 3, illustrating the scalp distribution of the amplitude difference between conditions.

**Figure 4:**
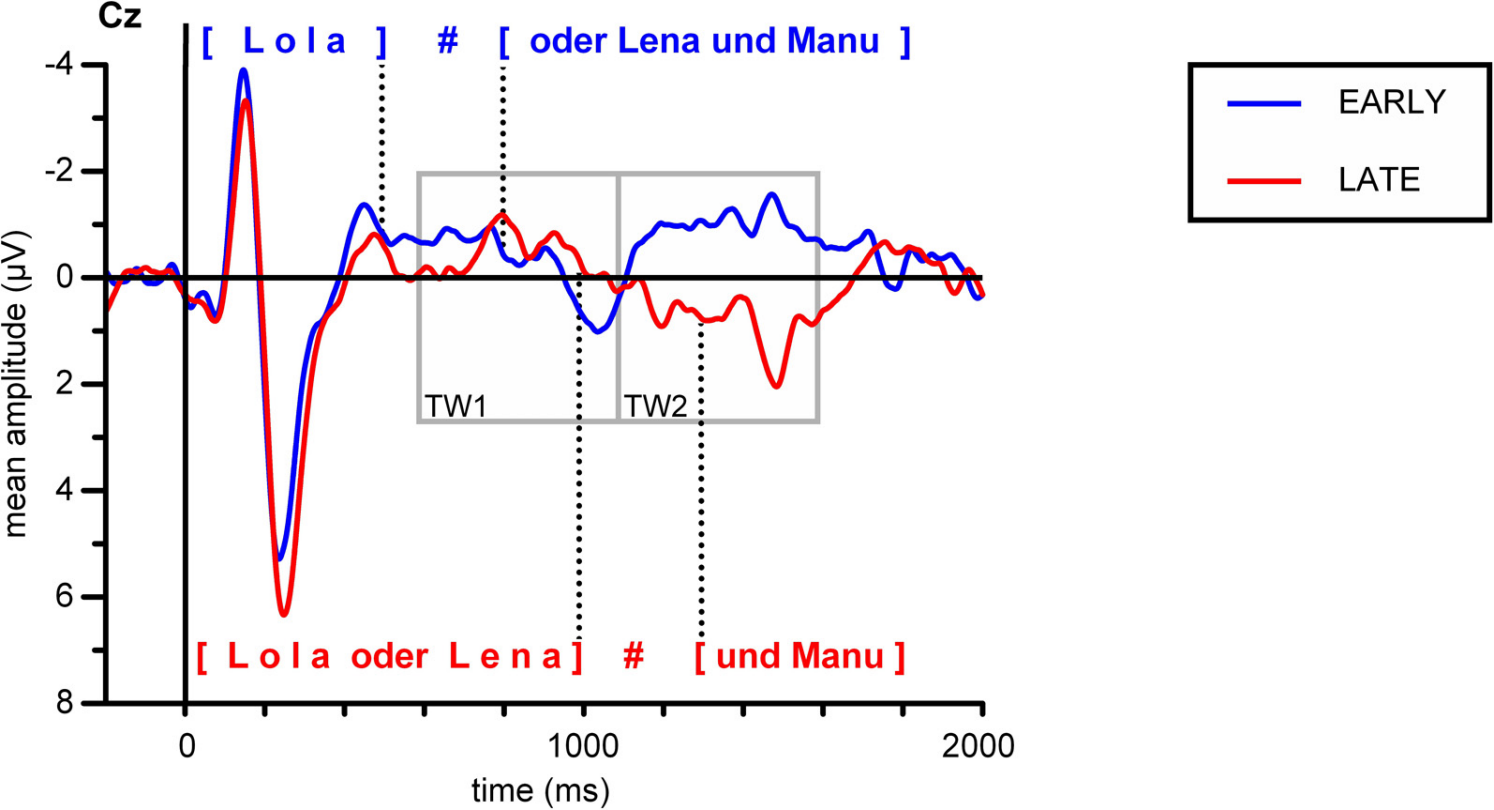
**Grand average ERPs for both conditions at electrode Cz**. Gray boxes indicate the time windows used in the statistical analysis relative to stimulus onset. Dotted lines indicate the mean onset and offset of the pause at the IPB in the respective condition, the silent pause is indicated by a hash mark.

**Figure 5:**
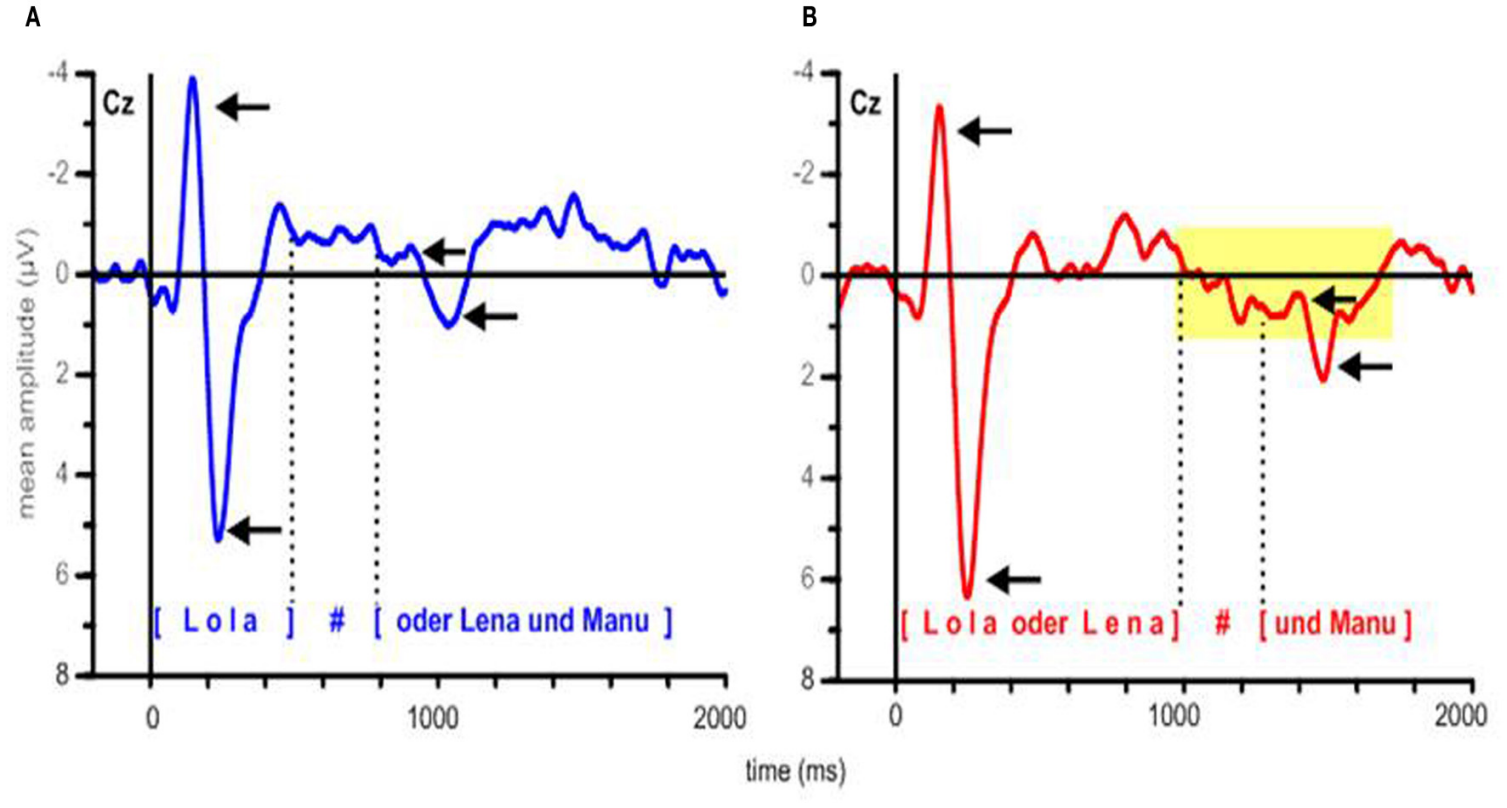
**Grand average ERPs at electrode Cz, depicted separately for (A) the EARLY and (B) the LATE condition**. Arrows indicate the N100 and P200 components at the stimulus onset and at the onset of the second part of the utterance. Dotted lines delimit the mean pause intervals; the silent pause is indicated by a hash mark. In panel **(B)**, the yellow rectangle indicates the time interval in which a positive shift can be observed, starting with pause onset and lasting for approximately 700 ms.

In Figure 4, grand average ERP responses at the representative Cz electrode are displayed. The dotted lines mark the mean pause interval in the two conditions, which lasted from ~500 to 800 ms after stimulus onset in the EARLY condition and from ~1000 to 1300 ms in the LATE condition. For both conditions, the obligatory N100-P200 complex (part of the auditory evoked potential, AEP; see, e.g., Picton et al., 1974) is evoked in response to the stimulus onset from ~100 to 300 ms. Moreover, ERPs in both conditions display this obligatory components in response to the onset of the second part of the utterance after the pause. Here, the N100-P200 complex is less pronounced, presumably because it reflects a new onset within the utterance (as compared to utterance-initial) and because the pause duration slightly varies over stimuli (see Table 2). Still, a clear combination of a negative deflection followed by a positive peak can be found within the first 300 ms after pause offset—that is, around 1000 ms after stimulus onset in the EARLY condition and around 1500 ms in the LATE condition (to the right of the respective pause intervals indicated in Figure 4). To illustrate the onset components, the grand average ERP waves to each condition are depicted separately in Figures 5A,B.

In addition to the obligatory components, a broad positive deflection can be observed for the LATE condition. It starts with the end of the first utterance part at around 1000 ms and lasts for ~700 ms (see Figures 4, 5B). In the EARLY condition, a corresponding positive deflection that coincides with the offset of the first utterance part should start at around 500 ms after stimulus onset (see Table 2). As can be seen in Figures 4, 5A, no such broad positivity is present in the EARLY condition. Hence, a positive shift coinciding with the IPB can only be observed for the LATE condition.

#### Statistical analyses relative to stimulus onset

For the statistical analysis relative to stimulus onset, epochs of 2000 ms were adjusted to a prestimulus baseline of 200 ms. Two consecutive time windows of 500 ms were defined in line with the possible occurrence of a CPS in response to the two experimental conditions. Remember that the IPB position in the EARLY condition, that is, the offset of the first name, was on average at 488 ms after stimulus onset, while in the LATE condition, the IPB occurred at the end of the second name, on average 992 ms after stimulus onset. Given these different IPB positions in the stimulus material, a CPS in response to the IPB in the EARLY condition should be revealed by statistical analyses of the first time window (TW1, 600–1100 ms after stimulus onset), while a CPS in response to the IPB in the LATE condition should lead to an effect of condition in the second time window (TW2, 1100–1600 ms).

A fully crossed repeated measures ANOVA was computed with the factors Time window (TW1 vs. TW2), Condition (EARLY vs. LATE boundary), Region (anterior, central, posterior), and Hemisphere (left vs. right); participants were entered as a random factor. The same analysis was conducted for the midline electrodes except that instead of the factors Region and Hemisphere only the factor Electrode (Fz vs. FCz vs. Cz. vs. Pz) was included. Subsequently, significant interactions involving the factor Condition were further analyzed using ANOVAs involving the respective factors. Only significant amplitude differences involving the factor Condition are reported. Where appropriate, a correction according to Greenhouse and Geisser (1959) was applied and reported as the corrected significance.

For lateral sites, the ANOVA including the factors Time window, Condition, Region and Hemisphere revealed a statistically significant interaction of Time window × Condition [*F*_(1, 17)_ = 13.25, *p* < 0.01]. For midline electrodes, an ANOVA including the factors Time window, Condition and Electrode revealed a significant interaction of Time window × Condition × Electrode [*F*_(3, 51)_ = 4.31, *p* < 0.05] and a significant interaction of Time window × Condition [*F*_(1, 17)_ = 12.45, *p* < 0.01].

To test the interaction with the factor Time window, subsequent statistical analyses were carried out on each time window separately. For both time windows a One-Way ANOVA with the factor Condition was computed for lateral sites and a Two-Way ANOVA including the factors Condition and Electrode for the midline electrodes.

For the first time window (600–1100 ms), neither at lateral nor at midline sites was a significant main effect of Condition present, nor an interaction of Condition × Electrode at the midline electrodes, suggesting no differences between conditions at the early boundary position. For the second time window (1100–1600 ms) a statistically significant main effect of Condition was present for lateral electrode sites [*F*_(1, 17)_ = 8.82, *p* < 0.01] as well as for the midline electrodes [*F*_(1, 17)_ = 6.72, *p* < 0.05] with mean amplitudes in the LATE condition being more positive than in the EARLY condition.

In sum, this analysis relative to sentence onset indicated the occurrence of a broadly distributed CPS corresponding to the IPB at the LATE boundary position, whereas in response to the IPB at the EARLY boundary position, no positive shift occurred.

**Figure 6:**
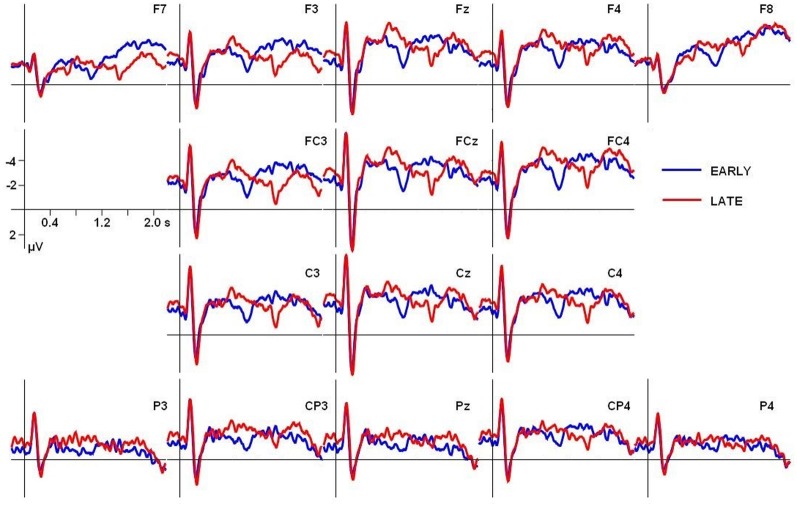
**Grand average ERPs for both conditions adjusted to a baseline from 200 to 400 ms after stimulus onset, covering stimulus initial onset components**.

However, one reviewer suggested additional analyses with a baseline of 200 to 400 ms after stimulus onset instead of a prestimulus baseline to compensate for differences in the ERP wave forms occurring early after stimulus onset (see Figure 4). Figure 6 depicts the grand average ERPs for both conditions, adjusted to the 200–400 ms baseline. Moreover, slightly different time windows were proposed to quantify the CPS effects, with TW1 ranging from 700 to 1150 ms and TW2 ranging from 1150 to 1600 ms after stimulus onset. Paralleling the initial analysis, two fully crossed repeated measures ANOVA were computed separately over lateral and midline electrodes, including the factors Time window (TW1 vs. TW2) and Condition (EARLY vs. LATE boundary); participants were entered as a random factor. The statistical analysis employing the new baseline and slightly different time windows revealed a statistically significant interaction of Time window × Condition for lateral [*F*_(1, 17)_ = 19.79, *p* < 0.001] as well as for midline electrodes [*F*_(1, 17)_ = 17.64, *p* < 0.001]. Subsequent statistical analyses testing the interaction with the factor Time window were carried out on each time window separately. In contrast to the previous analysis using a prestimulus baseline, for the first time window (700–1150 ms) a significant main effect of Condition was present at lateral [*F*_(1, 17)_ = 14.86, *p* < 0.01] and midline electrodes [*F*_(1, 17)_ = 10.82592, *p* < 0.01]. For the second time window (1150–1600 ms) a statistically significant main effect of Condition was only present for lateral electrode sites [*F*_(1, 17)_ = 4.82, *p* < 0.05] but failed to reach significance for midline sites [*F*_(1, 17)_ = 2.34, *p* = 0.1442325]. Hence, the additional analysis using a different baseline period and differing time windows does not support the findings from the initial analysis. Instead, it points at differences between conditions in both time windows with an even more pronounced effect in the early time window. Therefore, the statistical data analyses relative to sentence onset lead to inconclusive results depending on the choice of baseline period and/or time window.

This highlights the importance of a thorough ERP data quantification especially regarding the choice of baseline and time windows employed (see also Steinhauer and Drury, 2012). Special care has to be taken in the interpretation of auditory ERPs since they are susceptible to acoustic changes and latency differences in the stimulus material. First, the investigation of prosodic boundary processing virtually always comes with critical latency differences in the stimulus material, because noun phrases before an IPB are longer than noun phrases at non-boundary positions (due to final lengthening, see material section above). Time-locking the ERPs to the boundary position, for example to the offset of the noun phrase followed by an IPB, allows to compensate for these latency differences between conditions (see Kerkhofs et al., 2007; Bögels et al., 2010, for comparable analyses). This may especially be necessary if subsequent boundary positions occur within one stimulus as in the present study. Second, it is necessary to disentangle the CPS from the P200 component (see, e.g., Picton et al., 1974) in response to the speech onset after the IPB (see Steinhauer, 2003; Kerkhofs et al., 2007; Männel and Friederici, 2009; Pauker et al., 2011). The previously described analyses did not meet this requirement, because subsequent P200 components occur within the time windows chosen to quantify possible CPS effects, as can be seen in Figure 4 (but note that subsequent onset components should have equally affected latency differences for both the EARLY and the LATE condition). Apparently, at this point a more fine-grained data analysis is needed to conclusively quantify the observed effects.

Hence, additional analyses relative to potential boundary positions within the stimuli were conducted to meet this need and to be able to draw reliable conclusions regarding the presence of CPS effects in response to the stimuli presented.

### Analyses relative to NP offset

Additional analyses were conducted relative to the offset of the first name (or noun phrase, henceforth, NP1 offset), representing the early boundary position, and to the offset of the second name (NP2 offset), representing the late boundary position. Instead of the previous prestimulus baseline ERP epochs were now adjusted to a baseline of 50 ms prior to NP offset. This bears the additional advantages that (1) there is an equal distance between the baseline and the time window used for the statistical analysis at each boundary position under investigation and (2) the relatively short baseline prior to NP offset allows compensating for potential differences in the onset components characterizing the first 400 ms of stimulus processing (see Figure 4).

The time window for the statistical analyses relative to NP offset was defined as 100–300 ms after NP offset. Given that acoustic cues triggering boundary perception (that is, final lengthening and pitch change) are already available prior to the offset a time window starting 100 ms after NP offset should be suitable to evidence the positivity in mean amplitudes signaling IPB processing (see, e.g., Bögels et al., 2010; Pauker et al., 2011). Although CPS effects have been found to peak around 300 to 500 ms after NP offset (e.g., Bögels et al., 2010; Pauker et al., 2011), the time window chosen here ends earlier to avoid an influence of the subsequent P200. Given that pause duration ranges from 250 to 322 ms (see Table 2), it is obvious that the P200, a positivity peaking around 200 ms after pause offset, cannot be held responsible for amplitude differences found within the chosen time window.

Separate ANOVAs were conducted for lateral and midline electrode sites including the same topographical levels as in the analysis relative to stimulus onset. Instead of the factor Time window and Condition, the analyses now contained the factors Position (that is, either NP1 offset or NP2 offset) and Boundary (boundary status, either with or without IPB) with two levels each. All significant amplitude differences involving the factors Position and/or Boundary are reported and significant interactions with these factors were further analyzed with separate ANOVAs. Where appropriate, a correction according to Greenhouse and Geisser (1959) was applied and reported as the corrected significance.

For lateral sites, an ANOVA including the factors Position, Boundary, Region and Hemisphere revealed a statistically significant interaction of Position × Boundary × Region × Hemisphere [*F*_(2, 34)_ = 9.69, *p* < 0.01], as well as significant interactions of Position × Boundary × Region [*F*_(2, 34)_ = 3.83, *p* < 0.05], Position × Hemisphere [*F*_(1, 17)_ = 5.33, *p* < 0.05] and Position × Region [*F*_(2, 34)_ = 7.13, *p* < 0.01]. For midline electrodes, an ANOVA including the factors Position, Boundary and Electrode revealed a significant interaction of Position × Boundary × Electrode [*F*_(3, 51)_ = 5.41, *p* < 0.01] and a significant interaction of Boundary × Electrode [*F*_(3, 51)_ = 12.87, *p* < 0.001].

To test the respective interactions, subsequent statistical analyses were carried out (1) for each position (that is, NP1 or NP2 offset) and (2) for each boundary status (that is, with or without IPB) separately.

ANOVAs for lateral and midline electrode sites for the early position (NP1 offset) did not reveal significant effects involving the factor Boundary, apart from an interaction of Boundary × Region × Hemisphere for the lateral sites [*F*_(2, 34)_ = 4.96, *p* < 0.05]. Since subsequent two-way ANOVAs for each level of Region and Hemisphere did not reveal any effects for the factor Boundary, this effect was disregarded. Thus, additional statistical analyses suggested no differences between stimuli with and without an IPB at the early position (offset NP1).

In contrast, subsequent ANOVAs at the late position (NP2 offset) revealed main effects of Boundary at lateral sites [*F*_(1, 17)_ = 6.45, *p* < 0.05] and midline electrodes [*F*_(1, 17)_ = 5.61, *p* < 0.05], as well as interactions of Boundary × Region [*F*_(2, 34)_ = 12.83, *p* < 0.001] and Boundary × Electrode [*F*_(3, 51)_ = 25.68, *p* < 0.001], respectively. Hence, a clear difference between stimuli with and without an IPB is present at the late position (NP2 offset).

To determine the topographical position of this effect, subsequent One-Way ANOVAs were conducted for each region (lateral sites) and accordingly electrode (midline) testing the aforementioned interactions. Significant main effects of Boundary were revealed for the lateral electrodes at anterior [*F*_(1, 17)_ = 10.34, *p* < 0.01] and central [*F*_(1, 17)_ = 8.67, *p* < 0.01] regions and at the midline electrodes Fz [*F*_(1, 17)_ = 15.71, *p* < 0.01] and FCz [*F*_(1, 17)_ = 9.86, *p* < 0.01], suggesting a fronto-central distribution of the CPS observed for the IPB at the late boundary position.

Regarding the boundary status, ANOVAS for lateral and midline electrode sites for epochs containing no boundary cues [that is, without IPB, either at the early (NP1 offset) or late (NP2 offset) position] did not show significant effects involving the factor Position, apart from an interaction of Position × Region × Hemisphere for the lateral sites [*F*_(2, 34)_ = 4.41, *p* < 0.05]. Since subsequent ANOVAs for each level of region and hemisphere did not reveal any effects for the factor Position, this effect was disregarded. Thus, this control comparison suggested no relevant differences between the early position and the late position when no IPB is present.

Crucially, significant differences were obtained comparing epochs with IPB at the early position (NP1 offset) and at the late position (NP2 offset): at lateral electrode sites, a main effect of Position [*F*_(1, 17)_ = 7.18, *p* < 0.05] and an interaction of Position × Region [*F*_(2, 34)_ = 3.97, *p* < 0.05] were present. Analyses for midline electrodes revealed a marginally significant effect of Position [*F*_(1, 17)_ = 4.37, *p* = 0.05183] and an interaction of Position × Electrode [*F*_(3, 51)_ = 5.46, *p* < 0.05]. Hence, the direct comparison between ERPs in response to an IPB at the early position and to an IPB at the late position confirmed the difference between the conditions found in the initial analysis relative to stimulus onset.

To further determine the topography of this difference, subsequent ANOVAs were conducted testing the interactions of Position × Region (lateral sites) and accordingly Position × Electrode (midline). Significant main effects of Position were revealed for the lateral electrodes at anterior [*F*_(1, 17)_ = 8.15, *p* < 0.05] and central [*F*_(1, 17)_ = 9.57, *p* < 0.01] regions and at the midline electrodes Fz [*F*_(1, 17)_ = 9.30, *p* < 0.01] and FCz [*F*_(1, 17)_ = 6.91, *p* < 0.05], supporting the notion of a fronto-central distribution of the CPS effect.

Taken together, the additional statistical analyses confirmed the effect suggested by the initial broader analysis relative to sentence onset (with a prestimulus baseline) but differ from the analysis with a baseline covering the obligatory components of stimulus onset: For the ERPs time-locked to the offset of the critical NPs, statistical differences between the EARLY and the LATE condition were only obtained at the late boundary position (NP2 offset), whereas no differences were present at the early boundary position (NP1 offset). Moreover, epochs with IPB significantly differed as a function of the boundary position, whereas no such amplitude difference could be found for the respective epochs without IPB. Subsequent analyses resolving interactions with the factor Region revealed a fronto-central distribution of the CPS effect observed for the IPB at the late boundary position.

## Discussion

Here, we tested whether the occurrence of the CPS depends on the position of the IPB in the stimuli. In both conditions, the IPB was clearly signaled by three acoustic cues, namely a pitch rise, final lengthening, and a pause. The conditions only differed in regard to the position of the IPB: in the EARLY condition, the IPB already occurred after the first in a list of three names, while in the LATE condition, the boundary occurred after the second name. The results showed that a typical CPS is only elicited in response to a late IPB. When the IPB occurred early in the stimulus material, however, no positive shift was observed. Hence, we found a positional effect which demonstrates that the occurrence of a CPS-like pattern depends on contextual factors such as the position of a prosodic boundary within an utterance.

Given that the occurrence of the CPS indicates prosodic phrase boundary processing we suppose that the prosodic cues, which were unequivocally present in both conditions, were processed in different ways, depending on their position in the utterance. For the LATE condition, the interpretation is straightforward: In line with previous ERP research on prosodic boundary processing (e.g., Steinhauer et al., 1999, see above), the CPS occurs as a marker of IPB processing. The fronto-central scalp distribution matches previous CPS findings. Though the topography of the CPS varies to some extent over studies—presumably depending on the stimuli used (see, e.g., Pannekamp et al., 2005 for different scalp distributions depending on the material used)—CPS effects have been reported not only with a broad distribution (e.g, Steinhauer et al., 1999; Kerkhofs et al., 2007), but also with a fronto-central distribution (e.g., Itzhak et al., 2010). Interestingly, Pannekamp et al. (2005) also found a fronto-central maximum of the CPS when they tested participants with so-called jabberwocky sentences. Since the only content words in the stimuli used here were six proper names, it may well be that the stimuli were processed in a comparable way as stimuli without semantic content, but with appropriate function words and morphemes. Moreover, the latency of the obtained CPS effect is in line with previous studies, where the positive shift has been described to start almost immediately with the end of the preboundary utterance part (i.e., after the onset of the pause; see, e.g., Bögels et al., 2010; Itzhak et al., 2010; Pauker et al., 2011) and to last around 500–700 ms (e.g., Pauker et al., 2011, see also above).

Notably, previous ERP studies always used long sentences as stimulus material to investigate prosodic boundary processing. To our knowledge, the current study is the first that demonstrates that the CPS can also be elicited for boundaries in short, non-sentential sequences. Since behavioral studies (e.g., Lehiste, 1973; Wagner, 2005; Kentner and Féry, 2013) have also used this kind of coordinate structure to investigate prosodic phrasing in production and perception, this finding is further evidence for the CPS as an indication of prosodic boundary processing.

In the EARLY condition, two analyses (time-locked to stimulus onset with (1) a prestimulus baseline and (2) a baseline covering stimulus initial onset components) came to differential results, see above. As mentioned earlier, the investigation of prosodic boundary processing virtually always comes with critical latency differences in the stimulus material. Therefore, we conducted a more sophisticated analysis time-locked to the boundary position (offset of critical NP) in addition to the time-locking to stimulus onset that allows to (a) compensate for these inherent latency differences between conditions and (b) disentangle the CPS from post-boundary onset components (P200). This analysis confirmed the absence of a CPS in response to the IPB right after the first word (NP1). This is surprising because the boundary cues did not differ in strength from the cues that were present in the LATE condition. As the CPS generally occurs whenever a major prosodic boundary is processed—independent of the segmental, lexico-semantic or syntactic content (Pannekamp et al., 2005), we assume that the prosodic cues that were present in the EARLY condition—pitch rise, final lengthening, and pause—were not effectually used for prosodic phrasing and hence did not elicit a CPS as in the LATE condition. In other words, we do not find an effect of online boundary processing in the EARLY condition, because the prosodic changes seem not to be interpreted as cues to an IPB. How can this difference in processing be explained? It is assumed here that the crucial difference between the ERP patterns in the EARLY and the LATE condition (and, importantly, also between our EARLY condition and the stimulus material used in previous research on the CPS) lies in the shortness of the first IP. How can we account for an influence of phrase length on the processing of the boundary cues? In the following, two possible lines of argumentation will be sketched.

Firstly, the prosodic changes in the EARLY condition may not have been processed as cues to an IPB because there was not enough previous prosodic information available to evaluate them as IPB cues. This reasoning would be in line with behavioral studies demonstrating an influence of the magnitude of a previous prosodic boundary on boundary perception (e.g., Carlson et al., 2001; Clifton et al., 2002; Wagner and Crivellaro, 2010). Remember that these authors argue that prosodic boundary cues are always processed relative to other, previously processed boundaries. In the EARLY condition of our study, no such benchmark is available to the listener when the IPB is encountered, whereas in the LATE condition, in contrast, a weaker prosodic boundary (signaled by the moderate pitch rise at the end of the first name) has already been processed once the IPB occurs. Moreover, as prosodic information is not only available at boundary positions but unfolds over time, the previously processed prosodic context in general may serve as a reference system for boundary perception. Our results would hence in addition reflect a length effect: The processing of local boundary cues relies on previously processed prosodic information, which in our case unfolds during the perception of the longer constituents in the LATE condition. In the EARLY condition, no such or not enough previous contextual information (e.g., information on segmental duration and pitch variation) is available to interpret the prosodic boundary cues as such during online processing. This length effect seems at first glance inconsistent with the behavioral results of Clifton et al. (2006). Remember that the authors found a larger effect of boundary perception for stimuli with short constituents as compared to stimuli with long constituents and argued that boundaries after short constituents are more informative to the listener. In contrast, we found an effect of boundary processing only at the late boundary position, when a longer constituent precedes the boundary. However, this contrast may be easily explained by the differences in the experimental design. Clifton et al. (2006) ascribe their finding to the fact that after long constituents a prosodic break may be inserted for reasons irrelevant to syntactic parsing (i.e., speech fluency). As the coordinate structures we used were in general rather short—even in the LATE boundary condition the first constituent consisted of no more than five syllables—this reasoning does not necessarily hold for our material and thus it may not be appropriate to expect the type of length effect Clifton et al. (2006) describe. Hence, despite the reversed direction of the length effect, our results are in accordance with the findings of Clifton and colleagues and could either mirror a mere length effect or be interpreted in line with the findings on the impact of relative prosodic boundary strength (see above). Accordingly, our results are consistent with a non-local account for prosodic boundary processing assuming a context-dependent interpretation of prosodic boundary cues.

Secondly, the missing CPS in the EARLY condition may be due to an unnecessity for chunking at the early boundary position. Prosodic boundaries enable the listener to chunk the incoming auditory signal into larger units and may hence help to reduce processing costs and to guide the parser. Remember that in the EARLY condition, the boundary cues are encountered when listeners have only perceived a minor part of the utterance (in fact, only two syllables, i.e., the first proper name). Therefore, there may simply be no need to chunk this word into a larger (prosodic) unit—a cognitive process that may be reflected by the CPS. This would imply that the significance of the CPS goes beyond the pure detection or encounter of prosodic boundary cues. In fact, our data support the idea that the CPS is not only mirroring perceptual processes. Rather, it has a linguistic or cognitive relevance signaling the use of prosodic boundary information during online processing. Note that this reasoning holds for the notion of unnecessary chunking as well as for the suggested account of lacking prosodic context.

Crucially, one has to keep in mind that the initial analysis time-locked to sentence onset with a baseline controlling for differences in onset components indicate the presence of a more positive going ERP at the point of the IPB also in the EARLY condition. This highlights the susceptibility of ERP analyses to the choice of baseline and time window parameters especially in speech processing and the necessity of a proper stimulus design that allows for a time-locking to the critical events in the speech stream.

Future research is necessary to clarify under which conditions a CPS may be elicited even at the first boundary position or after short IPs. For example, one could gradually enlarge the first noun phrase by using polysyllabic names or by adding a determiner or a modifying adjunct. However, at least in the latter case one has to keep in mind that adding more material to the IP may lead to an additional (weaker) prosodic boundary. With the material used in our study we clearly cannot disentangle if the contextual information necessary to elicit a CPS is purely prosodic in nature or whether also additional syntactic or lexico-semantic information may add to the visibility of a CPS in the EARLY condition. Given the findings of Pannekamp et al. (2005) we speculate that linguistic domains other than prosody have a minor influence on the CPS. However, due to the absence of a specific task in our experimental setting, it may well be that listeners did not entirely process the syntactic structure of the material. A task forcing participants to resolve the syntactic ambiguity may hence lead to enhanced CPS effects. Potentially, this could imply the occurrence of a CPS in the EARLY condition.

The present study yields two pieces of evidence for the incremental processing of prosodic information. Firstly, the immediate integration of prosodic boundary cues is reflected by the CPS elicited in the LATE condition. Secondly, contextual prosodic information may also have an immediate influence on the processing: in the EARLY condition the use of prosodic boundary cues seems not be warranted by the preboundary context, either due to missing benchmark prosodic information or because it is not necessary from a cognitive resource viewpoint. Therefore, no CPS-like ERP pattern occurs. This shows, in turn, that the occurrence of a CPS does not reflect the brain's response to acoustic changes which may indicate an IPB, but rather that it mirrors the integration of available prosodic boundary information into the parsing process, that is, it signals the use of prosodic boundary cues for sentence comprehension. In conclusion, we have shown that a CPS is not necessarily elicited whenever the relevant prosodic boundary cues are present. Instead, the occurrence of the CPS was influenced by the IPB position, which was correlated with differences in the length of the preceding constituent and in the occurrence of an earlier boundary in the stimulus material. Further research is needed to determine the exact nature of the apparent impact on CPS occurrence. The result can be interpreted in line with a non-local account of boundary processing, because previously processed information has an immediate impact on the processing mechanism. In addition, by using electrophysiology we find evidence for an immediate integration of prosodic cues into the parsing of an utterance as long as this is affirmed by the previously processed context. Regarding the functional relevance of the CPS, this study yields further evidence that the CPS does not reflect pure signal detection, but rather mirrors the use and integration of prosodic boundary information during online spoken language comprehension.

### Conflict of interest statement

The authors declare that the research was conducted in the absence of any commercial or financial relationships that could be construed as a potential conflict of interest.
